# Occupational health and safety regulations in China: development process, enforcement challenges, and solutions

**DOI:** 10.3389/fpubh.2025.1522040

**Published:** 2025-04-07

**Authors:** Xinyu Xu, Peihua Ren, Wenyan Shi, Feidan Deng, Qingfeng Wang, Shaoteng Shi, Jingchun Shen, Guoqiang Dong, Jing Han

**Affiliations:** ^1^Institute of Hygiene for the Arms Industry, Xi'an, Shaanxi, China; ^2^Global Health Institute, Health Science Center, Xi'an Jiaotong University, Xi'an, Shaanxi, China; ^3^Department of Occupational and Environmental Health, School of Public Health, Health Science Center, Xi'an Jiaotong University, Xi'an, Shaanxi, China; ^4^Key Laboratory for Disease Prevention and Control and Health Promotion of Shaanxi Province, Xi'an Jiaotong University, Xi'an, Shaanxi, China; ^5^Environmental Science Academy of Shaanxi Province, Xi'an, Shaanxi, China

**Keywords:** occupational health, preventing and controlling difficulties, legislative system, future needs, occupational safety challenges

## Abstract

**Background:**

Occupational health is closely related to China’s public health system and the construction of a healthy China. The purpose of this paper is to summarize the current health status of China’s occupational population and the dilemmas faced by prevention and treatment work.

**Methods:**

The following databases were utilized: China National Knowledge Infrastructure (CNKI), Wanfang Data, PubMed, Web of Science, Scopus, and Google Scholar. Keywords used included “occupational health,” “occupational hygiene,” “occupational disease prevention,” “occupational risks,” “occupational health regulations,” and “China.” The search was limited to articles published from 2000 to the present to ensure the inclusion of the most recent studies and regulatory updates.

**Results:**

It is found that changes in industrial structure have led to significant differences in the production environment and labor practices of different occupations, which in turn have triggered the gradual emergence of various types of occupational diseases. The existing legislative system of occupational diseases protects the rights and interests of workers to a certain extent, but there are still some shortcomings. The main dilemmas faced by the prevention and control work include the complexity and diversity of occupational disease hazards, the lack of implementation of prevention and control measures, and the imperfection of the legislative system.

**Conclusion:**

This study proposes directions and measures for good prevention and control of occupational diseases, and explores the road of prevention and control of occupational diseases with Chinese characteristics. By improving the legislative guarantee, optimizing the implementation mechanism, and deepening the social co-governance, a sustainable occupational health management system adapted to the needs of industrial upgrading will eventually be constructed.

## 1 Introduction

Occupational health and safety (OHS) regulations refers to the laws and regulations adopted in protection of the safety and health of workers in the production process (including improving working conditions, preventing industrial accidents and occupational hazards, practicing the combination of work and rest, and the special protection of female workers and underage workers). It is critical components in safeguarding the well-being of workers and ensuring a sustainable and productive workforce (1). In China, rapid industrialization and economic development over the past few decades have brought about significant changes in the occupational landscape, with the country becoming more deeply integrated into the global division of labor system, relying on its advantages in terms of land and labor to become the “world’s factory” (2). However, while rapid economic growth has improved the overall quality of life and life expectancy of the population, it has also posed potential risks to OHS (3, 4).

Factors affecting occupational diseases cover multiple interacting domains, including the work environment, occupational hazards, and labor rights and protection (5, 6). The state is usually responsible for taking these actions. However, occupational diseases are characterized by the co-existence of multiple disease threats and the intersection of multiple health influences (7). Traditional industries such as mining and manufacturing still pose significant occupational health threats, while emerging industries such as technology and services have introduced new occupational hazards (8). Susan et al. showed that prolonged use of computers and other electronic devices may lead to Computer Vision Syndrome. Psychosocial factors are a category of occupational risk factors that have recently attracted the attention of researchers (9, 10). Rudo M S Chingono et al. analyzed the psychological stress of medical work during the new crown and they experience a high burden of mental health symptoms (11).

In addition, individuals’ background and level of knowledge before exposure to OHS regulations may influence their understanding and acceptance of these regulations (12, 13). In addition, people from different occupational backgrounds may focus on different aspects of regulations to determine their applicability and effectiveness (14, 15). For the government, the traditional regulatory model emphasizes access, prior licensing, and supervisory coverage. In the context of the reform of the administrative approval system and the further deepening of the clearance of professional qualifications, it is clear that further emphasis on access, prior permission. However, with the rapid development of the economy, the volume of enterprises is large and changing rapidly, while the supervision team is limited. To achieve full coverage of occupational health supervision for all enterprises, the existing strength of the regulatory team is far from adequate. Although relevant results have been reported in different multidisciplinary studies, they are often hidden as control variables or of secondary research interest. Systematic reviews on corporate tax evasion and the effectiveness of government regulation are still missing. Failure to take these factors into account may limit the effectiveness and enforceability of regulations.

By synthesizing the available evidence and exploring the links between exposure to certain information characteristics and regulatory understanding and enforcement, this review provides theoretical support and practical guidance for the future development of OHS regulations. Given the limited theory and knowledge on the topic, we consider all types of information characteristics, including information content, sources, and channels. Based on the available empirical evidence, we provide a roadmap for future research.

## 2 Methods

### 2.1 Search strategy

In this research, our search strategy was completed and executed on February 12, 2024. We utilized China National Knowledge Infrastructure (CNKI), Wanfang Data, PubMed, Web of Science, Scopus, and Google Scholar as our primary databases. The refined search strategy emerged from an ongoing refinement of key terms identified in relevant papers, incorporating terms such as “occupational health, ““occupational hygiene, ““occupational disease prevention, ““occupational risks,” “occupational health regulations,” and “China.” To enhance the precision of our literature retrieval, we employed Boolean operators in constructing the search query. Additionally, we confined our search to articles published between 2000 and November 3, 2024 to ensure the inclusion of the latest research findings and regulatory changes. Perform the literature search by the above strategy and apply the following screening criteria: (1) exclude documents that are not pertinent to the subject matter, retaining only those categorized as “papers, reviews, and online publications”; (2) restrict the language of the articles to English and Chinese, discarding documents in other languages.

### 2.2 Quality assessment

**Figure 1:**
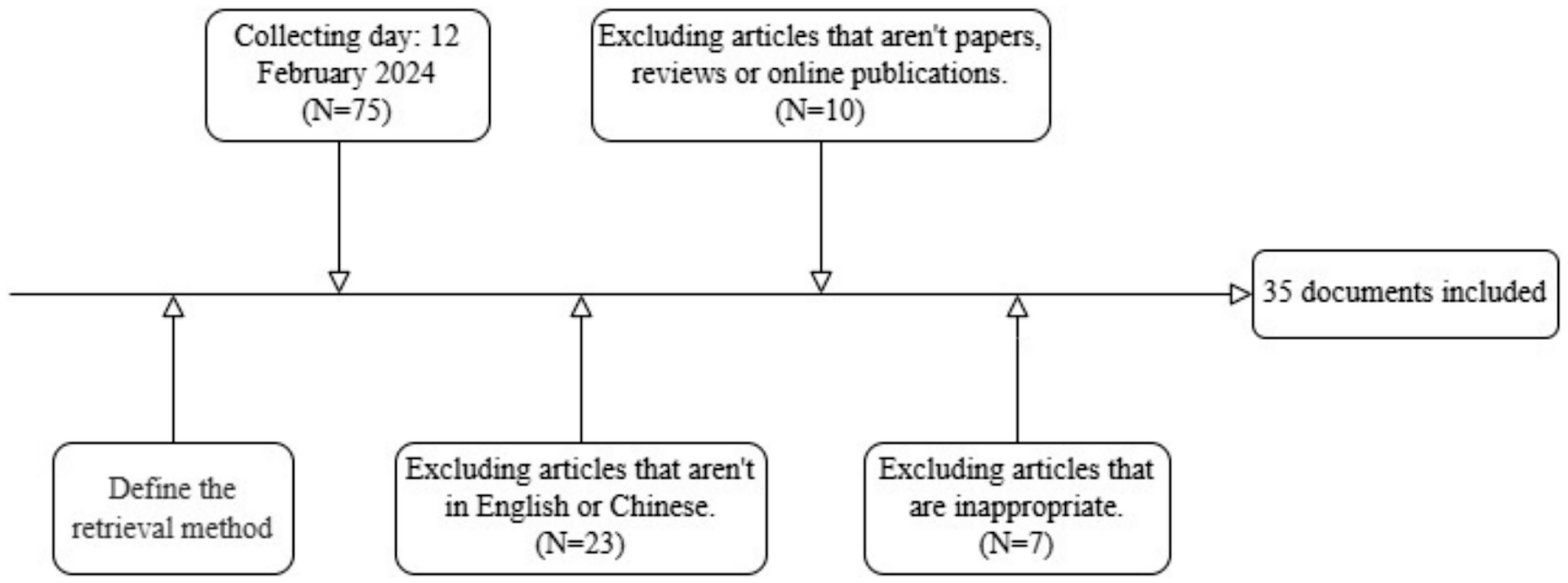
Flow diagram of literature search and study selection process.

The methodological quality of each study included in the review was independently assessed by two authors (SWY and RPH). We used CASP research evaluation tool—consideration based on assessment dimensions, including clarity of research purpose, methodological soundness, and data credibility (16). Quality was assessed through a list of 14 items, with a score of a maximum 14. A study with a score of less than 5 was considered to be of poor quality, a score between 5 and 9 as of fair quality, and greater than 9 as of good quality. Discrepancies were discussed until a consensus was reached, with assistance from the senior author (HJ), if necessary. In cases of ambiguity, discussions were held with additional authors involved in the project. Ultimately, 35 documents were selected for inclusion (Figure 1). The flowchart depicted in Figure 1 illustrates the entire process of study search and selection.

## 3 Health status of the occupational population in China

*China’s large labor force base determines the importance of occupational health issues, and the following data clearly shows the current health status of the working population.* As of 2017, 776 million people were employed in China accounting for 55.8% of the total population. Most workers spend more than half of their lives in their professional careers (17). By the end of 2022, there were 18.08 million reports of occupational health check cases in China, including 328,000 cases of occupational contraindications and 12,000 suspected occupational diseases (18). According to the recently released data from the 2022 Seventh National Census, China’s population has exceeded 1.41 billion, with 509 million living in rural areas and 901 million in urban areas. The estimated percentage of the working-age population is 63.35% (19, 20). Over 60% of the population is engaged in the service sector, while 355 million people work in the secondary industry (Primarily refers to industry, which is the industrial sector that reprocesses primary products, including mining, manufacturing, and construction), accounting for 35.5% (21). Many of these individuals work in high-risk industries for occupational diseases, such as chemicals, mining, asbestos, and non-ferrous metals.

The main occupational risks include accidental injuries, pneumoconiosis (especially silicosis), musculoskeletal injuries, chronic obstructive pulmonary disease, pesticide poisoning, alcohol poisoning, asbestosis, noise-induced hearing loss, and workplace stress. In 2017, there were 323,800 deaths in China attributable to exposure to these harmful occupational factors (95% UI: 283,800 to 369,100), with 14,060,210 (ranging from 12,022,974 to 16,125,753) disability-adjusted life years attributable to these occupational hazards (22). The increasing proportion of women in the labor market exacerbates traditional occupational disease issues. Due to biomass fuel, physiological conditions, and the dual burden of household and professional work, the diagnosis age and exposure duration for female patients with asbestosis are lower than those for men (23, 24).

According to the Health and Wellness Statistical Bulletin 2021, there were 15,407 reported cases of occupational diseases in 2021 (25). However, due to difficulties in diagnosing and identifying certain occupational diseases without relevant legal determinations, the official case numbers may be underestimated (26). It is clear from these data that occupational health problems involve many workers, especially in high-risk industries, and that the situation of prevention and control is grim.

## 4 The dilemma of occupational disease prevention and control in China

### 4.1 The industrial structure determines the basic occupational hazard situation

With the continuous adjustment and transition of industrial structure, the diversification of employment systems, and the complexity of operational methods, new occupational health issues have emerged. The development and application of new chemical substances, and the introduction of new processes and technologies, all bring new challenges to occupational health. In addition, the adjustment and upgrading of manufacturing industry such as information technology, deep-sea operations, and aerospace operations have also triggered new occupational health risks (27). Shift work, overtime work, occupational stress caused by high-stress levels, as well as ergonomic issues such as fatigue, overwork, and musculoskeletal disorders resulting from improper work postures or behaviors, demonstrate that workers are facing increasing occupational health risks (28). With the development of socio-economics and changes in labor supply–demand relationships, the mobility of workers is increasing, and the management of mobile workers’ health has always been a difficulty and focus of occupational disease prevention and control. China is the country with the highest number of female workers in the world, mainly engaged in manufacturing and labor-intensive industries, where occupational health hazards are severe (29). As the retirement age extends, ensuring the health of older adult workers has become an important occupational health issue in developed countries and will also be a challenge that China needs to face in the future. If these issues are not properly addressed, they will impact the sustainable development of China’s economy.

### 4.2 Defects in the treatment of new pollutants

Recently emerging or attention-grabbing pollutants, known as new pollutants, possess characteristics of the ecological hazard, environmental persistence, and ecological accumulation (Table 1). They pose significant risks to human life safety or the natural environment without comprehensive environmental governance policies in place to control their risks (30). There are four main categories of new pollutants that have attracted widespread attention domestically and internationally: persistent organic pollutants, endocrine disruptors, antibiotics, and microplastics (31). In China, these new pollutants are widespread and exist at high concentrations, showing a trend of increasing concentrations from the western regions to the eastern regions. The geographical distribution of these four types of pollutants has their characteristics: persistent organic pollutants are mainly found in industrial prosperous areas in central, southwestern, and northern China, primarily originating from discharged industrial wastewater; endocrine disruptors are more common in industrially and agriculturally developed coastal areas; antibiotics have a relatively broad distribution, being discovered in water bodies and sediment nationwide, with sulfonamides mainly distributed in inland river lakes and quinolones predominantly found in coastal areas; microplastics are mostly carried by rivers and drift, ultimately primarily distributed in the southeastern coastal regions (32).

**Table 1:** Emerging pollutant types, characteristics and hazards.

Type	Analysis methods	Main sources	Disposal methods	Impacts
Persistent Organic Pollutants (POPs)	Gas Chromatography-High Resolution Mass Spectrometry (GC–HRMS), Gas Chromatography–Mass Spectrometry (GC–MS)	Industrial production, pesticides, firefighting foam, electronics production	High-temperature incineration, chemical degradation, biodegradation	Strong bioaccumulation, difficult to degrade, can lead to cancer, immune system damage, and various reproductive issues
Endocrine Disruptors (EDCs)	Liquid Chromatography-Mass Spectrometry (LC–MS), Enzyme-linked Immunosorbent Assay (ELISA)	Industrial chemicals (such as Bisphenol A), personal care products, certain medications, and pesticides	Activated carbon adsorption, ozonation, advanced oxidation processes	Mimic or interfere with normal hormone function, affecting the reproductive system and development, potentially leading to obesity, diabetes
Antibiotics	Liquid Chromatography–Tandem Mass Spectrometry (LC–MS/MS), High-Performance Liquid Chromatography (HPLC)	Medical wastewater, livestock farming, aquaculture	Biological treatment technologies, adsorption technologies, advanced oxidation technologies	Enhances bacterial resistance, poses a threat to environmental microbial communities, impacts human health
Microplastics	Microscopic examination, Fourier Transform Infrared Spectroscopy (FTIR), Raman Spectroscopy	Fragmentation and degradation of plastic products, cosmetics, laundry detergents	Mechanical filtration, biodegradation, chemical degradation	Bioaccumulation, impacts aquatic organism health, potentially affects human health through the food chain

To more effectively manage new types of pollutants, China has introduced a series of policies and governance measures. In November 2021, the Communist Party of China Central Committee and the State Council issued the “Opinions on Deepening the Tough Battle Against Pollution Prevention and Control.” Subsequently, in May of the following year, the State Council General Office released the “Action Plan for the Governance of New Pollutants.” At the same time, in December 2022, the Ministry of Ecology and Environment, along with five other ministries, jointly issued the “Key Controlled New Pollutants List (2023 Edition).” China’s goals and key focus areas for the governance of new pollutants have been clearly defined, and various regions have successively introduced relevant work plans to advance the governance of new pollutants. However, the current management of new pollutants in China is still in its early stages (33). Issues such as legislative gaps in addressing new pollutants generated under the backdrop of the times and industrial development (including endocrine disruptors, persistent organic pollutants like perfluoro compounds, antibiotics, and microplastics), insufficient experience in prevention and control, weak government supervision, lack of adequate understanding and awareness among workers and related enterprises, the use of hazardous chemicals harmful to the environment and humans in production processes, and ineffective measures to reduce emissions of new pollutants persist. Compared to traditional pollutants, new pollutants are more complex in toxicity, less biodegradable, and pose higher risks to human health. Their deposition in production processes and the environment severely impacts the health of China’s workforce (34).

### 4.3 The responsibilities of all parties are not well implemented

#### 4.3.1 The employer fails to perform its statutory obligations

According to Article 47 of the “Occupational Disease Prevention and Control Law,” employers have the responsibility to truthfully provide workers’ occupational history, exposure to occupational disease hazards, workplace hazard detection results, and other necessary information for diagnosing and identifying occupational diseases. Health administrative departments should supervise and urge employers to provide the mentioned information (5). However, in China’s current process for handling occupational diseases, which includes determining labor relations, diagnosing occupational diseases, recognizing work-related injuries, and assessing labor capacity, some non-compliant companies frequently set up obstacles at each step (35). They refuse to provide crucial information such as workers’ occupational history and exposure to occupational disease hazards, impeding the diagnosis and identification of occupational diseases. By neglecting or purposely delaying the signing of labor contracts, they prevent workers from proving their labor relationship with employers, hindering work-related injury recognition. These actions significantly obstruct the effective resolution of occupational disease issues and harm the rights and health of workers.

### 4.4 The infrastructure for prevention and control of occupational diseases is not sound

**Figure 2:**
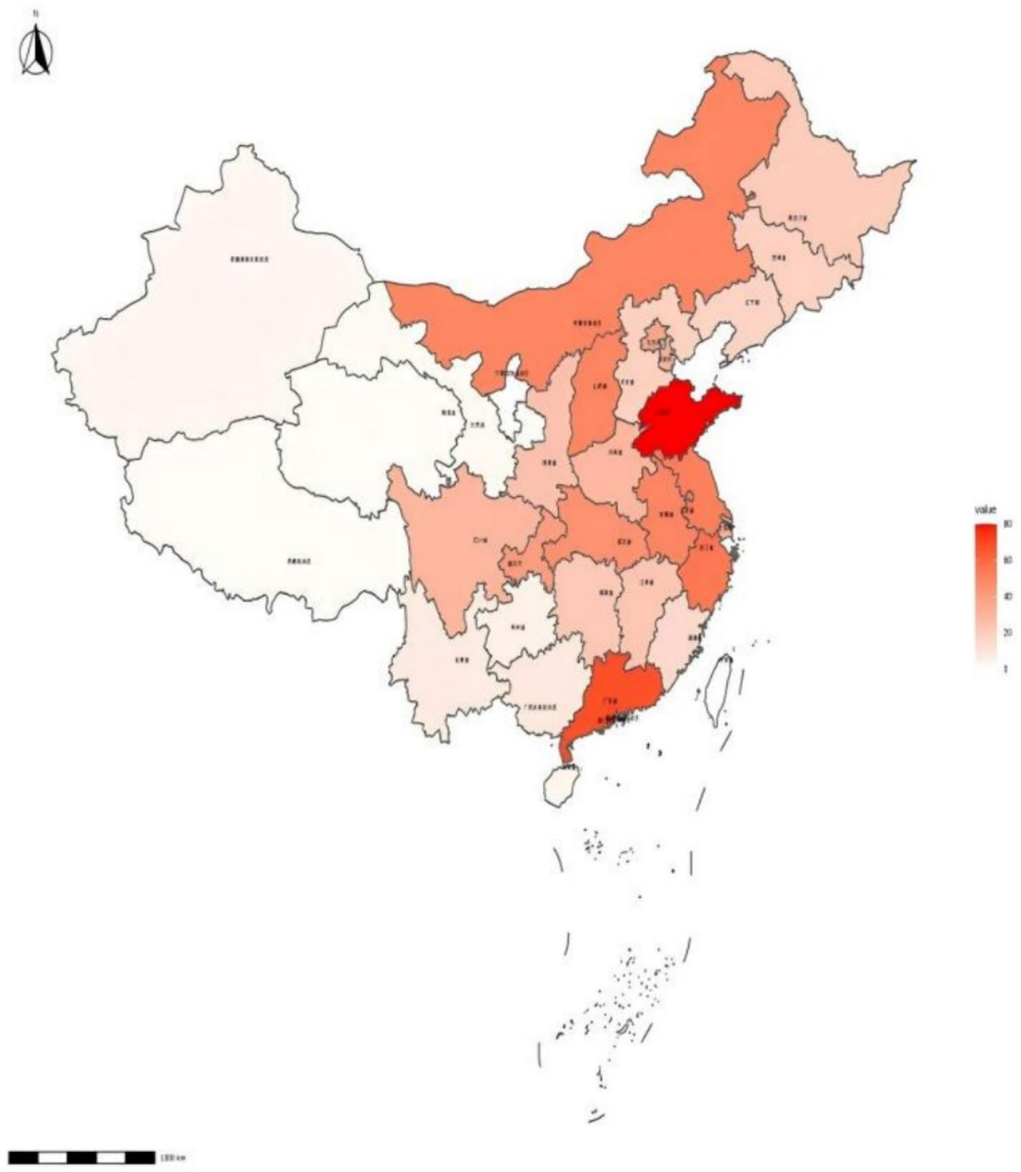
The distribution density of occupational disease diagnosis and healthcare service institutions in China. Shades of red represent the number of occupational health facilities in the location. The darker the red color, the higher the number of occupational health organizations.

The number of health service institutions specializing in diagnosing occupational diseases in China is limited and unevenly distributed, leading to difficulties in diagnosing such diseases. The “Occupational Disease Prevention and Control Law” sets qualification requirements for institutions involved in the diagnosis and identification of occupational diseases. Only hospitals with the necessary diagnostic qualifications can provide definitive diagnoses for occupational diseases. Additionally, only diagnostic certificates issued by hospitals with identification qualifications have legal validity. As of 2021, China has a total of 1,022 occupational health technical service institutions, 605 radiation health technology service institutions, 23 centers for chemical toxicity identification, 5,067 employee health examination institutions, and 588 occupational disease diagnosis institutions. This means that approximately 34,000 high-risk workers have only one institution available for diagnosis (36) (Figure 2). Although the number of institutions is of a certain size, the average number of high-risk workers, service resources are still insufficient, and the inadequate infrastructure for occupational disease prevention and control significantly hinders the effective implementation of occupational disease prevention and control efforts. Insufficient diagnostic services mean that many occupational disease patients cannot promptly detect and diagnose their conditions, missing the optimal treatment window, leading to disease progression, and even irreversible health damage. Moreover, late-stage diagnosis of occupational diseases increases patients’ treatment costs, exacerbates their financial burden, and has a more significant impact on families and society as a whole.

### 4.5 Workers have a weak awareness of protecting their rights

The majority of workers in China have a weak understanding of legal rights and lack awareness of how to protect their rights through legal means. From a judicial perspective, the group most at risk of developing occupational diseases tends to be lower-income and less-protected migrant workers. Due to the concealed and delayed nature of occupational diseases, workers may not exhibit symptoms upon leaving their jobs. Additionally, these workers often have limited knowledge of legal matters and weak awareness of their rights due to disparities in social resource allocation. Although laws like the “Occupational Disease Prevention and Control Law” clearly stipulate the obligation of employers to provide relevant information and specific burden of proof responsibilities, many workers do not know how to retain the necessary evidence during the process of diagnosing occupational diseases or recognizing work-related injuries. Some may not even have labor contracts with their employers, leading to a series of arbitration and litigation processes to determine their labor relationship (37). This difficulty in providing evidence and protecting rights makes it challenging for some occupational disease patients to pursue their rights, often resulting in them giving up on seeking justice.

### 4.6 Differences in stakeholder perspectives

Employers generally express concerns about the high costs associated with implementing strict occupational health measures, especially for small—and medium—sized enterprises. They argue that excessive regulations may limit their competitiveness. However, workers emphasize the importance of their health rights and demand better protection. They often face difficulties in obtaining information from employers and lack awareness of how to safeguard their rights. Regulatory authorities, on the other hand, point out the challenges of insufficient resources and unclear division of responsibilities in supervision, which makes it difficult to ensure full compliance.

## 5 Current situation and deficiency of occupational health legislation in China

### 5.1 Current status of occupational health legislation in China

Currently, China has established a comprehensive legal and regulatory framework. This system is centered around the “Occupational Disease Prevention and Control Law,” with three key regulations as its backbone—namely, the “Pneumoconiosis Prevention and Control Regulations,” the “Regulations on Labor Protection in Workplaces Using Toxic Chemicals,” and the “Regulations on Radiation Protection for Radioactive Isotopes and Radiation Devices.” It is supplemented by over 10 supporting regulations such as the “Classification and Catalog of Occupational Diseases,” the “Management Measures for Occupational Health Examinations,” the “Catalog of Classification of Occupational Hazard Factors,” and more than 430 national occupational health standards (GBZ series) (38) (Table 2).

**Table 2:** Historical development of the law in the People’s Republic of China on prevention and control of occupational diseases.

Time	Regulations
1950	Draft Regulations on Factory Hygiene
1957	Provisions on the Scope of Occupational Diseases and Methods for Handling Occupational Disease Patients
1982	Trial Measures for Reporting Occupational Poisoning and Occupational Diseases
1983	Revision of “Measures for Reporting Occupational Poisoning and Occupational Diseases” to “Measures for Reporting Occupational Diseases”
1984	Decision on Strengthening Dust and Poison Prevention Work
1987	Management Measures for Occupational Disease Diagnosis
1988	Revision of Provisions on the Scope of Occupational Diseases and Methods for Handling Occupational Disease Patients
2002	Regulations on the Labor Protection of Female Workers
2016	Occupational Disease Prevention and Control Law
2017	Regulations on Labor Protection in Workplaces Using Toxic Substances
2018	First Amendment to the Occupational Disease Prevention and Control Law

The “Occupational Disease Prevention and Control Law of the People’s Republic of China” has been in effect since 2002. Subsequently, the Standing Committee of the National People’s Congress amended this law four times in December 2011, July 2016, November 2017, and June 2018 to adapt to new administrative and economic situations. The latest revision entails a realignment of the administrative roles of government institutions. Generally, the State Administration of Work Safety manages occupational hazards in workplaces, while the National Health Commission manages worker health. In the new revision, the legal responsibilities of the State Administration of Work Safety were removed, with oversight and management of occupational disease prevention left to the health and labor departments of the people’s government. The declaration of occupational disease hazards and classification directories are now formulated solely by the State Council’s health administrative department, concentrating authority to prevent interdepartmental buck-passing and delays, ensuring clearer responsibilities, and enabling relevant departments to swiftly address occupational hazard issues. However, this has led to challenges such as a noticeable decrease in the technical proficiency of personnel handling occupational health supervision and management tasks (39). This law delineates the responsibilities of employers, employees, government institutions, authorized occupational health service providers, and other stakeholders, aiming to protect employees from the impact of occupational diseases. If companies violate the law, the health administrative department can impose fines of up to 500,000 RMB and may even require businesses to suspend operations for rectification.

### 5.2 Inadequacy of occupational health legislation in China

#### 5.2.1 Occupational health supervision function is absent

In China, the occupational health regulatory system has undergone three major reforms as recorded. The current system being implemented is the centralized and unified regulatory system that has been in place since 2018. Following the revision of the “Occupational Disease Prevention and Control Law” in 2018, the health administrative departments and labor security administrative departments at or above the county level (referred to collectively as the occupational health supervision and management departments) are responsible for the supervision and management of occupational disease prevention and control according to their respective division of responsibilities. The health administrative departments are responsible for implementing occupational disease prevention and control work in employing units, managing occupational health technical service institutions, and occupational disease diagnosis, and identification, among other tasks. The labor security departments are responsible for the recognition of labor contracts, labor relations, work injury insurance, disability identification, and related matters. While this move has achieved specialized management and enabled each department to be responsible for different areas of work, fully leveraging their respective professional advantages, there are problems such as overlapping and ambiguous responsibilities, as well as uneven enforcement efforts. For example, there is a close connection between occupational disease diagnosis and work injury insurance; however, they fall under different departments. This division of responsibilities may lead to unclear responsibilities in practical operations, especially in cases that require both departments to handle them jointly. Differences in resource allocation and enforcement efforts among different departments may result in inadequate occupational disease prevention and control work in certain areas, potentially impacting the continuity and effectiveness of occupational safety supervision (40). Through the development of a harmonized health and safety directive, the EU has clarified the regulatory responsibilities of member states to reduce cross-functionality and improve enforcement efficiency. China can learn from it to optimize its regulatory system (41).

#### 5.2.2 The punishment of employing units is relatively low

Between 2018 and 2022, fines for OHS violations accounted for only 0.03% of industrial output in Guangdong Province—a figure 10-fold lower than the EU average (Guangdong Statistical Yearbook, 2023; Eurostat, 2023)—reflecting systemic under-prioritization of worker safety (42, 43).

Article 72 of the “Occupational Disease Prevention and Control Law” stipulates that “if an employing unit violates the provisions of this Law and engages in any of the following behaviors, the health administrative department shall issue a warning, order correction within a specified period; failure to correct within the given time frame may result in a fine ranging from fifty thousand to two hundred thousand RMB. In cases of severe circumstances, the order to stop operations causing occupational disease hazards shall be issued, or the relevant people’s government shall be requested to close down by the authority prescribed by the State Council (5).” The primary focus is on administrative penalties. Only when an enterprise generates unforeseeable serious consequences will it be ordered to close? However, these serious adverse outcomes often signify irreversible and irreparable damage to workers, even if the business is shut down, it may not achieve the fundamental goal of preventing occupational diseases through legislation (44). The relatively small fine amount may be perceived as part of operating costs by some companies and may not create a sufficient deterrent effect. Driven by economic interests, businesses might overlook occupational disease prevention measures, continuing to maintain working conditions that endanger the health of workers. Furthermore, the lack of specific time constraints for “correction within a specified period” in legal regulations leaves workers exposed to occupational disease hazards during this period, hindering the timely protection of workers’ health rights and interests.

#### 5.2.3 The basis for the construction of regulations is not comprehensive

The “Occupational Disease Prevention and Control Law” stipulates that for suspected cases, diagnosis of occupational diseases must be conducted by qualified doctors authorized by designated hospitals. The diagnosis must be based on: (1) the worker’s employment records, (2) qualified records of qualitative and quantitative exposure to occupational hazards in the workplace, and (3) clinical manifestations and test results of auxiliary examinations. To guide the implementation of this work, the Ministry of Health revised the “Regulations on Occupational Disease Diagnosis and Identification” based on the first edition issued on March 28, 2002, on January 9, 2013 (45). On January 4, 2021, the National Health Commission issued Order No. 6, announcing the latest “Management Measures for Occupational Disease Diagnosis and Identification.” In China, the formulation of regulations is primarily based on industry and elements. Most regulations are classified according to different matters, with relatively fewer regulations based on hazard element classification. At the level of standard formulation, while there is some overlap between industry classification and element classification standards, industry classification standards hold a numerical advantage. Even regulations and standards developed based on industry or elements do not fully encompass all aspects of occupational disease prevention. Such fragmented standards have adverse effects on practical operations (46).

#### 5.2.4 The occupational health monitoring system is not perfect

The health status of workers can change with variations in working conditions, making it crucial for employing units to monitor these changes through regular occupational health examinations and management of health records. This not only helps in understanding the health status of workers but also serves as critical evidence in resolving labor disputes. However, in reality, many employing units only conduct health checks before hiring, neglecting ongoing health supervision, leading to ineffective prevention of occupational hazards. This issue partly stems from the lack of specific timeframes in current Chinese laws and regulations regarding the regular health checks required for workers, and the specialized supervision by regulatory bodies over labor protection measures in employing units is lacking (41). Companies where workers are employed often do not prioritize occupational health protection adequately and overlook the requirement for proper management of health records, resulting in the ineffective establishment of workers’ health records. The discrepancy between the health status records of workers and the information known by health administrative departments hampers effective supervision of employing units and timely punishment for illegal activities. Therefore, there is a need for occupational health regulatory authorities to enhance oversight to ensure that employing units effectively implement the occupational health surveillance system (47).

#### 5.2.5 The scope of mandatory constraint is not clearly defined

Compared to developed countries like the United States and the European Union, the mandatory scope of enforcement for occupational health regulations and standards in China is not clearly defined. This ambiguity deepens the difficulties faced by enforcement agencies and companies in implementation, thereby reducing the efficiency of enforcing regulations and standards (48, 49). There are significant shortcomings in China’s occupational health regulatory system. Although labor laws stipulate that OHS work fall under the jurisdiction of labor administrative departments, companies with occupational disease hazards are supervised by health administrative departments, and safety production is managed by safety supervision departments, the actual division of regulatory responsibilities is not clear. With the transfer of responsibilities from safety supervision departments to the health commission, the lack of regulatory regulations has led to an unclear delineation of enforcement powers. This structural problem is equally prevalent in developing countries, such as India (50). Health administrative departments lack enforcement authority in the field of safety production, making it challenging to hold parties accountable for accidents effectively. Additionally, while multiple administrative bodies participate in occupational health supervision, theoretically capable of integrating resources to safeguard workers’ rights, in practice, health administrative departments lack independent accident investigation and technical support, hampering their ability to fully fulfill their duty of protecting workers’ occupational health rights.

#### 5.2.6 There are shortcomings in the path of judicial assistance for workers’ right to health at work

The primary way the country upholds workers’ occupational health rights is through the compulsory work-related injury insurance system. However, this compulsory insurance system has some shortcomings, particularly in strengthening assistance for workers’ rights and livelihood relief. One aspect of this deficiency refers to the relatively vague complementarity between the insurance system for work-related injuries and the civil compensation system, as well as the absence of a comprehensive criminal punishment system for upholding workers’ occupational health rights. China has a low per capita work-related injury insurance coverage rate due to high costs associated with work-related injury identification, difficulties in determining liability under new forms of employment, lack of uniform standards for recognizing occupational diseases, and cumbersome administrative procedures for work-related injury identification (51).

## 6 Future needs of occupational disease control

### 6.1 Raise awareness of occupational disease prevention in enterprises and workers

China urgently needs to establish awareness of occupational disease health among all stakeholders, including legislators, employers, employees, contractors, and the general public. Occupational disease health should be incorporated into educational curricula at all levels of schools, universities, and technical education institutions. Public awareness of the health hazards of environmental pollution and diseases that may arise from exposure to harmful substances should be raised through mass media such as the short-video platform TikTok. Unions can effectively prevent accidents and work-related illnesses by negotiating workplace safety and health as part of collective bargaining issues and exerting pressure on political leadership. Governments and enterprises can cooperate on annual occupational health training programs, and the frequency of training can be increased for enterprises in high-risk industries.

### 6.2 Develop infrastructure and capacity

China currently faces a serious shortage of occupational disease diagnoses and healthcare professionals. Most doctors lack training in occupational health, leading to a scarcity of skills in diagnosing and preventing occupational diseases. Currently, there are only a little over 9,700 trained and qualified occupational disease diagnostic physicians in China, which is far from sufficient to meet the demand (52). There are issues of regional imbalance in the distribution of occupational disease diagnostic institutions, and the technical levels of these qualified diagnostic medical institutions vary, collectively lacking the necessary proficiency to meet the demands of occupational disease prevention and control work (53, 54). China is challenged with integrating occupational health with general health services and providing occupational health services from medical schools and hospitals. Therefore, both the government and private sector should design and implement short-term courses to enhance doctors’ understanding and abilities in diagnosing occupational diseases. Additionally, graduate courses in occupational health and industrial hygiene need to be introduced, along with the establishment of high-level academic positions to enhance the teaching and research capabilities of universities in the field of occupational health.

### 6.3 Improve the occupational disease-related laws and regulations

Following Japan’ s 2004 OHS reforms, workplace fatalities dropped by 37% within 5 years—a trajectory achievable in China through similar legislative upgrades (55). China’s current occupational health laws, regulations, and technical guidelines are in place, but they require further improvement in terms of comprehensiveness and systematic structure. This includes adopting a human-centric legislative concept to ensure that legislation is aimed at promoting the overall welfare of society rather than just economic development. It is important to provide legal protection for workers in emerging forms of labor and new work formats to ensure their occupational health rights are safeguarded, regardless of their employment arrangements. Furthermore, legislation should shift from traditional statutorily oriented occupational disease directions to a risk-oriented approach, emphasizing prevention and accountability while establishing uniform occupational health standards and protection mechanisms. These measures aim to construct a comprehensive and unified legal system to protect workers’ occupational health rights comprehensively. The protective system designates digital network platforms as the main contributors to work-related injury insurance under this mode of operation. This model introduces changes where workers in new employment formats perform their tasks independently on digital platforms. To some extent, this alters the traditional employment structure, where employers or entities entirely manage and uphold their workers’ labor rights. Digital internet platforms providing services are obligated to safeguard workers’ occupational health rights (56). For workers suspected of having occupational diseases, especially those with long latency periods such as pneumoconiosis, the absence of qualified exposure records hampers diagnosis. The state should establish a robust legal framework for occupational disease prevention and control, outlining responsibilities and obligations for all parties involved. More specific and detailed technical guidelines need to be developed to ensure that the diagnosis and prevention of occupational diseases are conducted scientifically and accurately. The role of supervision of enterprises should be brought into play, such as the establishment of an enterprise occupational health credit scoring system. Specifically for diseases with long latent periods, requirements for qualified records should be established to enable timely diagnosis and treatment. Moreover, enhancing monitoring and assessment of occupational exposures will provide more reliable grounds for diagnosing occupational diseases.

### 6.4 Strengthen regulatory and enforcement responsibilities regarding the right to occupational health

The reform of the occupational health supervision and law enforcement system aims to ensure the adaptability of occupational health supervision rights with the enforcement system, with the fundamental objectives being national interests and public interests. To safeguard the life and health levels of workers, the state and society should clarify the responsibilities of health authorities and strengthen the guardianship of workers’ health rights. Clarifying the supervisory responsibilities of each health administrative department helps protect workers from occupational injuries and provides legal remedies. To enhance the efficiency of occupational health supervision, it is recommended to establish specialized negotiation mechanisms, enhance the legal expertise of occupational disease law enforcement personnel, and improve the completeness of hardware equipment. Additionally, strengthening the oversight of laborers’ occupational health protection by the health committee ensures the protection of workers’ health rights. Employers should strictly implement the occupational health inspection system and establish workers’ occupational health monitoring records. The government can establish specialized supervisory bodies to enhance oversight of employers. Lastly, health administrative departments should strengthen occupational health law enforcement, strictly investigate illegal activities, emphasize key points in protective work, and enhance the proactive nature of health supervision (57).

## 7 Limitations

This study has several noteworthy limitations. First, while the investigation provides valuable insights into the Chinese context, its geographically restricted scope may constrain the generalizability of findings to other national settings. Moreover, despite substantial regional disparities in economic development levels and regulatory enforcement mechanisms across China, our analysis did not comprehensively examine these geographical variations. To address these limitations, future research should consider adopting a dual-axis approach that incorporates both cross-national comparative frameworks and subnational regional analyses, complemented by updated longitudinal datasets to strengthen the validity and applicability of research outcomes.

## 8 Conclusion

China’s 14th Five-Year Plan advanced occupational disease prevention through multi-agency collaboration, yet new challenges persist: poor management of emerging pollutants, weak accountability, inadequate infrastructure, and low awareness of workers’ rights. Future efforts must prioritize awareness-raising campaigns, strengthen legal frameworks, and expand professional training. For traditional sectors (manufacturing/mining), real-time particulate monitoring should be mandatory. The construction industry need nationwide blockchain health surveillance. Gig economy platforms require legislation to enforce algorithm-driven workload/rest limits by amending the Occupational Disease Prevention Laws. Success hinges on tripartite collaboration: employers prioritizing health, workers engaging in prevention, and regulators optimizing oversight. Only through integrated action can China overcome these challenges and usher in a new era of occupational health.

## References

[1] Lucchini RG London L. Global occupational health: current challenges and the need for urgent action. Ann Glob Health. (2014) 80:251–6. doi: 10.1016/j.aogh.2014.09.006, PMID: 25459325

[2] Zhang KH. Urbanization and industrial development in China. In: Tang Z, editor. China’s urbanization and socioeconomic impact. Singapore: Springer Singapore (2017). 21–35.

[3] Pega F Momen NC Abubakar AHA al-Emam R Hassan MN Howard J. Monitoring workers’ health: focus on rights, determinants, and equity. Lancet. (2023) 402:1306–8. doi: 10.1016/S0140-6736(23)02049-4, PMID: 37838426

[4] Ran X Zhao Y. Behind the flexibility: insufficient occupational injury protection of gig workers in China. Front Public Health. (2023) 11:1117180. doi: 10.3389/fpubh.2023.1117180, PMID: 37139392 PMC10149918

[5] National People’s Congress of the People’s Republic of China. Law of the People’s Republic of China on Prevention and Control of Occupational Diseases. (2001). (Last revised 2018). Available online at: http://www.npc.gov.cn/

[6] Ruckert A Labonté R. The global financial crisis and health equity: toward a conceptual framework. Crit Public Health. (2012) 22:267–79. doi: 10.1080/09581596.2012.685053, PMID: 40101104

[7] Biao Z. Analysis of the situation of occupational disease prevention and control in the new era and suggestions for countermeasures Dis Surveill Control. (2019). 13: 433–435. Available online at: https://kns.cnki.net/nzkhtml/xmlRead/trialRead.html?dbCode=CJFD&tableName=CJFDTOTAL&fileName=JBJK201906006&fileSourceType=1&invoice=KyUrXsxW55UeGoLCgerESIJXgCETV8XioGPKbhSQGNaqXmjgBgOlMdxboiMoUPZvaju3AYVKiQ6awc8aFXD3DJYns7v92xkBNVz3UwxTgpzrxH6l4zzqt6FlceSm

[8] Acquadro Maran D Begotti T. A circle of violence: are burnout, disengagement and self-efficacy in non-university teacher victims of workplace violence new and emergent risks? Appl Sci. (2020) 10:4595. doi: 10.3390/app10134595

[9] Lalloo D Lewsey J Katikireddi SV Macdonald EB Demou E. Health, lifestyle and occupational risks in information technology workers. Occup Med (Lond). (2021) 71:68–74. doi: 10.1093/occmed/kqaa222, PMID: 33515462 PMC8034523

[10] Randolph SA. Computer vision syndrome. Workplace Health Saf. (2017) 65:328. doi: 10.1177/2165079917712727 28628753

[11] Chingono RMS Nzvere FP Marambire ET Makwembere M Mhembere N Herbert T. Psychological distress among healthcare workers accessing occupational health services during the COVID-19 pandemic in Zimbabwe. Compr Psychiatry. (2022) 116:152321. doi: 10.1016/j.comppsych.2022.152321, PMID: 35576673 PMC9055394

[12] Hu R Hu N Liu R Shi L Shi J Ling L. Association between occupational health and safety knowledge and behaviours among migrant workers: results from a cross-sectional study in China. BMJ Open. (2020) 10:e040143. doi: 10.1136/bmjopen-2020-040143, PMID: 33262190 PMC7709500

[13] Malin M Jaakkola N Luukkonen R Heloma A Lamminpää A Reijula K. Occupational health professionals’ attitudes, knowledge, and motivation concerning smoking cessation-cross-sectional survey. J Occup Health. (2020) 62:e12145. doi: 10.1002/1348-9585.12145, PMID: 32701202 PMC7377039

[14] Brønnum-Hansen H Foverskov E Andersen I. Occupational inequality in health expectancy in Denmark. Scand J Public Health. (2020) 48:338–45. doi: 10.1177/1403494819882138, PMID: 31763956

[15] Li S Chen H Huang X Long R. Who has higher willingness to pay for occupational safety and health?-views from groups with different public identities and differences in attention. Int J Environ Res Public Health. (2018) 15:15. doi: 10.3390/ijerph15081667, PMID: 30082646 PMC6121491

[16] Panesar A Gharanei P Khovanova N Young L Grammatopoulos D. Thyroid function during COVID-19 and post-COVID complications in adults: a systematic review. Front Endocrinol (Lausanne). (2025) 15:1477389. doi: 10.3389/fendo.2024.1477389, PMID: 39967901 PMC11832367

[17] State Council of the People’s Republic of China. Action for a Healthy China (2019–2030). (2019). Available online at: http://www.gov.cn/zhengce/2019-07/15/content_5409828.htm

[18] National Health Commission of China. 2022 National Occupational Disease Prevention and Control Report. Occup Health Emerg Rescue. (2023). 41:551. Available online at: https://d.wanfangdata.com.cn/periodical/zywsyyjjy202305005

[19] National Bureau of Statistics of China. Bulletin of the Seventh National Population Census. People’s Daily. (2021) 11. Available online at: https://www.stats.gov.cn/sj/zxfb/202302/t20230203_1901087.html?

[20] Shi P. China’s demographic imbalance and its adjustment strategy-analysis based on the data of the seventh national population census. Tax Econ. (2022) 1–10. Available online at: https://kns.cnki.net/reader/flowpdf?invoice=H51aF65MuA3RataaMOyDadwJiT4GJnN7AiKR2jWl6KXe5P+aih7lqRXsA7OfmbcT6y1YIf/W0t6NaWF5wNCZJv/5oP6YPorUt4cXNRbWQspnPQUNGV6wCvRr5oYOz2VXKOb5tamlIIGcvFnTRGUOmyOFgjJsCLk6T9bIttiZtq0=&platform=NZKPT&product=CJFQ&filename=SW

[21] Department of Population and Employment Statistics, National Bureau of Statistics of China. China Population and Employment Statistics Yearbook. Beijing. China: China Statistics Press (2021). 2021 p.

[22] Dai YF Zheng YX. Progress of research on occupational health and occupational diseases in China. Chin J Dis Control. (2022) 26:869–75. doi: 10.16462/j.cnki.zhjbkz.2022.08.001

[23] Xiaoming M Chunbo S Yue L Hongyan L Geng W Zhihua L. Characterisation of occupational hazards and occupational disease incidence among female workers in Qinghai Province from 2006 to 2016. Chin J Ind Med. (2017) 30:439–40. doi: 10.13631/j.cnki.zggyyx.2017.06.012

[24] Yanrong Q Yanfang Q Xian W. Analysis of 604 cases of female occupational diseases. China Public Health. (2005) 103. Available online at: https://kns.cnki.net/nzkhtml/xmlRead/trialRead.html?dbCode=CJFD&tableName=CJFDTOTAL&fileName=ZGGW200504064&fileSourceType=1&invoice=Tc0btRFCAJX/F7pssNIix2CwG3+QXuvPdV/+kjJYowm1vgCipFOxSvZBiExA9w48y304lSl1mqqd8jt+msu9zOwO7eUeZdNS/cJFuJtS7/gBbCkIIYxskFBI7gno

[25] National Bureau of Statistics of China. National Statistical Bulletin on Health and Health Care. (2021). Available online at: https://www.stats.gov.cn/sj/ndsj/2021/indexch.htm

[26] Shu DF. Problems and countermeasures: exploring the protection of workers’right to occupational health in China. J Shandong Univ. (2012) 125–129. Available online at: https://kns.cnki.net/nzkhtml/xmlRead/trialRead.html?dbCode=CJFD&tableName=CJFDTOTAL&fileName=SDZS201203020&fileSourceType=1&invoice=aTuuYbU0HT5u+DrvwvNP/M0aRgsZI/nxa6rsWqSifsDteabJrh/MNz3LEKuYgG/mCtlGF0gtjluiC7fqsqn0WfNK9weZCqKLdJOlD8Xbxvxt8RNN4+Lz2F7/AWli

[27] Biao Z. Analysis of the situation of occupational disease prevention and control in the new period and countermeasure suggestions. Dis Surveill. Control. (2019) 13:433–5. Available online at:https://kns.cnki.net/nzkhtml/xmlRead/trialRead.html?dbCode=CJFD&tableName=CJFDTOTAL&fileName=JBJK201906006&fileSourceType=1&invoice=KyUrXsxW55UeGoLCgerESIJXgCETV8XioGPKbhSQGNaqXmjgBgOlMdxboiMoUPZvaju3AYVKiQ6awc8aFXD3DJYns7v92xkBNVz3UwxTgpzrxH6l4zzqt6FlceSm

[28] Lems WF Anastasilakis AD Andreasen CM Paccou J Rolvien T Tencerova M. Basic and clinical scientists working together-do we make the best of both worlds? Calcif Tissue Int. (2025) 116:39. doi: 10.1007/s00223-025-01347-z, PMID: 39953279 PMC11828806

[29] Romero-Serrano R Arnaiz C Torres-Enamorado D Lancharro-Tavero I Arroyo-Rodríguez A. Occupational Health Injuries and Illness Among Women Workers in the Chemical Industry: A Scoping Review. Workplace Health Saf. Published online December 23, 2024. doi: 10.1177/21650799241302501 39713920

[30] Field JA Johnson CA Rose JB. What is ‘emerging’? Environ Sci Technol. (2006) 40:7105. doi: 10.1021/es062982z, PMID: 17180949

[31] Pei L Huimin H Tao Y Shiya C Junping B Hua D. Status quo, problems and management countermeasures of new pollutant pollution in China. Environ Monit Early Warn. (2022) 14:27–30+70. Available online at: https://kns.cnki.net/kcms2/article/abstract?v=uQzRnDzoTXG7uBE7LKsXHRLKf2fdCHyU9xQqdktSXBGwD6taInPcK8M_MfFL_m92zcDriqMmUpJT5s71mCopmaGNRKYUl1OQhLXY7Z8e-cTXDGY5lUZKSbpvgOPr-QQsXETyzHyNpHhLzj9kgiqvKUznjhJXubJLaYrOp41LGbkU0m2Wl5ywZw==&uniplatform=NZKPT&language=CHS

[32] Lin-jun ZHOU Meng-yuan LIANG De-ling FAN Wei-long XING Zhen WANG Wen GU. International Practices and Enlightenment for Environment Monitoring of Emerging Pollutant[J]. Journal of Ecology and Rural Environment. (2021) 37:1532–9. doi: 10.19741/j.issn.1673-4831.2021.0293

[33] Zhao L Zhao R. Ecological rule of law and enterprise green innovation – evidence from China's environmental courts. J Environ Manag. (2025) 374:124081. doi: 10.1016/j.jenvman.2025.124081, PMID: 39793496

[34] YingCui. Research on Legal Issues of Collaborative Treatment of New Pollutants in China [D] Beijing University of Chemical Technology (2024) doi: 10.26939/d.cnki.gbhgu.2024.001482

[35] Hong W. Dilemma of occupational disease rights protection and improvement of rights protection system. People’s forum. (2012) 17:78–9. doi: 10.16619/j.cnki.rmlt.2012.17.006

[36] Qiang QX. Drawing on the regulations and standards for prevention and control of occupational disease hazards in the United States. Labour Prot. (2017) 11:98–101. doi: 10.3969/j.issn.1000-4335.2017.11.034

[37] Yifei Z. The Predicament and Overcoming of Occupational Disease Rights Protection for Migrant Workers in China [D]. Nanjing Agricultural University. (2018). doi: 10.27244/d.cnki.gnjnu.2018.000043

[38] Zubing WANG Keyong LI Guang ZHENG Xuetao ZHANG Wuzhong LIU Hutao HUANG. Inheritance and development of occupational health in China[J]. Occupational Health and Emergency Rescue. (2019) 37:101–6. doi: 10.16369/j.oher.issn.1007-1326.2019.02.001

[39] Huang J He DZ. Development status of occupational disease prevention and control in China. Occup Health. (2022) 38:2140–6. doi: 10.13329/j.cnki.zyyjk.2022.0430

[40] Jianxiang L. Research on the Protection of Occupational Health Rights of Workers [D].Southwest Medical University. (2022). doi: 10.27215/d.cnki.glzyu.2022.000143

[41] Guangdong Provincial Bureau of Statistics. (2023). Guangdong statistical yearbook 2023_ [data report]. Available online at: http://stats.gd.gov.cn/.l

[42] Eurostat. (2023). Occupational health and safety fines in the EU (2018–2022)_[data set]. Available online at: https://ec.europa.eu/eurostat

[43] Jianguo L. On the Labor Law Protection of Occupational Health Rights [D] Xiamen University (2017).

[44] Yixing G. Research on the legal protection of the right to work environment under the perspective of occupational disease prevention Nanjing University of Aeronautics and Astronautics (2023). doi: 10.27239/d.cnki.gnhhu.2021.001697

[45] Regulations on Diagnosis and Identification of Occupational Diseases (no. 91). Available online at: https://www.gov.cn/gongbao/content/2013/content_2396616.htm

[46] Fangyan Z Xingchun W Li P. Problems and suggestions in occupational health regulation and related standards. Occup Health. (2012) 28:2410–6.

[47] Shangyuan Z. Occupational disease prevention and control and the relief of occupational disease patients’ rights. Southeast Acad. (2020) 2:143–52. doi: 10.13658/j.cnki.sar.2020.02.011

[48] Howard J. Occupational health issues in the USA. Occup Med. (2017) 67:2–4. doi: 10.1093/occmed/kqw162, PMID: 28057880

[49] Iavicoli S. The new EU occupational safety and health strategic framework 2014-2020: objectives and challenges. Occup Med (Lond). (2016) 66:180–2. doi: 10.1093/occmed/kqw010, PMID: 27016744

[50] Pingle S. Occupational safety and health in India: now and the future. Ind Health. (2012) 50:167–71. doi: 10.2486/indhealth.ms1366, PMID: 22790480

[51] State Council Information Office Regular Policy Meeting. (2019). Available online at: http://www.scio.gov.cn/xwfb/

[52] Ma YX Li T Yang KX. Understanding and application of the provisions on several issues concerning the trial of work injury insurance administrative cases. People’s Justice. (2014) 23:16–21. doi: 10.19684/j.cnki.1002-4603.2014.23.006

[53] Jianchao C Zhenxing L. Problems and countermeasures in quality management of occupational disease diagnostic institutions. Straits. J Prev Med. (2015) 21:75–6.

[54] Jun Z. Problems and countermeasures of occupational disease diagnosis and appraisal in Beijing. China. Occup Med. (2013) 40:155–6.

[55] Ministry of Health, Labour and Welfare (MHLW). (2021). Rōdō anzen eisei hakusho: Reiwa 2-nendo ban_ [annual report on labour safety and health: Fiscal year 2020].

[56] Qi Kexin Research on Legal Governance of Overwork (Master’s Thesis, Liaoning University) (2018). Available at: https://kns.cnki.net/kcms2/article/abstract?v=sFOWa -sRa0TlnPf24ZcIGzh5JS3Ej8FCEuJV0MMLX5gX7JcE1YdI61MB-cNW8jJZX3oGUFPE2dE54i144syQdea6o2nXI7dVP6cs1Hny9kChK9BTaSuaReTZVOUdkfMDB_s6RM0U-x4Ly82pwcSSK_aby1FKG-vecZJEzYfk2mCftiyrd0aEZPOnCDz6nYY6WrPiD3G1pZY=&uniplatform=NZKPT&language=CHS

[57] Shu DF. Problems and countermeasures:exploring the protection of labourers’ right to occupational health in China. J Shandong Univ. (2012) 3:125–9. Avilable at: https://kns.cnki.net/kcms2/article/abstract?v=sFOWa-sRa0Tzz87Zy5qea8w3ou0RIm1qCIWIDQGKVd8cHIMzPr_YPHjEt14TCetujDqFFhv72uB_eFuMZpA_BE-9jD3Ic5f4oz7K4PrUH7eGvk04DiukxogmBiB4PL8AZDFylkImSAnV0vE9W_AYsdZDJG9RkkU3WVA8SKMXZj5VV5sd15ZcIyAyWu4liXbk&uniplatform=NZKPT&language=CHS

